# Event-Related Potentials in Assessing Visual Speech Cues in the Broader Autism Phenotype: Evidence from a Phonemic Restoration Paradigm

**DOI:** 10.3390/brainsci13071011

**Published:** 2023-06-30

**Authors:** Vanessa Harwood, Alisa Baron, Daniel Kleinman, Luca Campanelli, Julia Irwin, Nicole Landi

**Affiliations:** 1 Department of Communicative Disorders, University of Rhode Island, Kingston, RI 02881, USA; barona@uri.edu; 2 Haskins Laboratories, New Haven, CT 06519, USA; daniel.kleinman@yale.edu (D.K.); irwinj1@southernct.edu (J.I.); nicole.landi@uconn.edu (N.L.); 3 Department of Communicative Disorders, University of Alabama, Tuscaloosa, AL 35487, USA; lcampanelli@ua.edu; 4 Department of Psychology, Southern Connecticut State University, New Haven, CT 06515, USA; 5 Department of Psychological Sciences, University of Connecticut, Storrs, CT 06269, USA

**Keywords:** event-related potentials, audiovisual, phonemic restoration, speech perception

## Abstract

Audiovisual speech perception includes the simultaneous processing of auditory and visual speech. Deficits in audiovisual speech perception are reported in autistic individuals; however, less is known regarding audiovisual speech perception within the broader autism phenotype (BAP), which includes individuals with elevated, yet subclinical, levels of autistic traits. We investigate the neural indices of audiovisual speech perception in adults exhibiting a range of autism-like traits using event-related potentials (ERPs) in a phonemic restoration paradigm. In this paradigm, we consider conditions where speech articulators (mouth and jaw) are present (AV condition) and obscured by a pixelated mask (PX condition). These two face conditions were included in both passive (simply viewing a speaking face) and active (participants were required to press a button for a specific consonant–vowel stimulus) experiments. The results revealed an N100 ERP component which was present for all listening contexts and conditions; however, it was attenuated in the active AV condition where participants were able to view the speaker’s face, including the mouth and jaw. The P300 ERP component was present within the active experiment only, and significantly greater within the AV condition compared to the PX condition. This suggests increased neural effort for detecting deviant stimuli when visible articulation was present and visual influence on perception. Finally, the P300 response was negatively correlated with autism-like traits, suggesting that higher autistic traits were associated with generally smaller P300 responses in the active AV and PX conditions. The conclusions support the finding that atypical audiovisual processing may be characteristic of the BAP in adults.

## 1. Introduction

Speech perception and processing in naturalistic settings often involve both auditory (heard) and visual (seen) information from a speaker. Speech and language development is said to take place within this audiovisual (AV) context [1,2,3,4]. Visible information from a speaking face, specifically visual access to the mouth, impacts speech processing and can improve speech understanding [5,6,7]. Thus, deficits in AV speech perception may negatively impact a burgeoning language system, contributing to language delays further along the developmental trajectory. 

Audiovisual speech perception has been studied in both neurotypical and clinical populations. One specific clinical population that demonstrates atypical AV speech perception is autistic individuals [8,9,10,11,12,13,14,15,16]. Collectively, studies on AV speech perception in autism reveal that both autistic children and adults demonstrate difficulties in the online, simultaneous processing of auditory and visual information and are often less influenced by visual information from a speaking face when compared to neurotypical counterparts. Although several studies have investigated audiovisual speech perception in autistic individuals, less is known regarding AV speech in the broad autism phenotype (BAP) [17,18]. The broad autism phenotype refers to first-degree relatives of individuals with autism spectrum disorder (ASD) that display subclinical behaviors associated with the disorder. Twin and family studies of ASD suggest that autism traits are highly heritable [19], with a milder expression of traits in siblings and parents who have a child with ASD. Furthermore, other dimensional approaches suggest that ASDs represent an upper extreme constellation of traits that are continuously distributed within the population [20,21,22]. An emerging body of evidence suggests that there is a relationship between AV speech perception and autism traits within the broader population [11]. Therefore, we will use the term broader autism phenotype (also noted as BAP) to refer to individuals with elevated autistic traits who may not be first-degree relatives of an autistic person. The investigation of the broader population warrants further exploration, as it could provide valuable information regarding the nature of AV speech in relation to social and linguistic skills, even for those who do not have a formal diagnosis. 

One means to study AV speech perception is through electrophysiological methods, which allow for sophisticated measurement of the neural mechanisms associated with the processing of differing auditory and visual signals. Electroencephalography (EEG) and specifically event-related potentials (ERPs) measure neural activity that is time locked to a stimulus. Given that speech is a rapidly changing acoustic signal that unfolds over time, ERPs allow for the sophisticated measurement of AV speech processing within the temporal domain to determine differences which may arise when speakers are presented with auditory-only contexts compared to when speech is coupled with visible articulatory information. 

Broadly speaking, ERP components can be characterized as exogenous (<300 ms) or endogenous components (>300 ms). Exogenous components that occur prior to 300 ms are reported to reflect neural processing related to the sensory stimulus. These components can be recorded in passive listening conditions and do not require an overt response from the participant. The auditory N100 component is an exogenous component in linguistic research that has been shown to reflect the properties of the acoustic stimulus (i.e., loudness, duration, etc.) [23]. The N100 component is a negative ERP response occurring ~100 ms post-stimulus onset and recorded in frontocentral electrodes that can be recorded during passive conditions [24]. Studies that have investigated audiovisual speech perception in adults have revealed that the N100 response is modulated by audiovisual conditions, and different sensory information may impact the latency and or amplitude of the N100 component. Specifically, the auditory N100 may be attenuated [25,26,27] or decreased in latency [25,26,28] when paired with the visible articulation of speech. Endogenous components reflect the higher-order processing of the stimulus and are less determined by the physical features of the stimulus. The P300 ERP component is a highly studied endogenous component used in linguistic research. The P300 is a rather large positive deflection within the parietocentral region that peaks at ~300 ms (up to 900 ms following the stimulus), irrespective of the stimulus type (visual or auditory) or response requirements (physical button press or mental counting). The P300 is widely believed to be the neural representation of the mechanisms involved in attention and working memory [29]. The P300 component can also be modulated by different experimental conditions such that the amplitude of the P300 response can increase when greater attention is allocated to the task [29,30].

Our team has been using ERPs to study AV speech perception within a specific paradigm which exploits the phonemic restoration effect [31,32,33] and elicits both the N100 and P300 response. Phonemic restoration takes place when visual stimuli of speech (e.g., video of a speaker producing the syllable /ba/) is paired with an absent or degraded auditory stimulus (in this case, the energy in the /b/ consonant /ba/ is reduced, creating the auditory percept /*a*/). The visual features of the speaking face can “restore” the consonant, essentially facilitating the perception of /ba/ for the listener (Irwin et al.) [2,11,34]. Irwin et al. [11] investigated the neural correlates of audiovisual speech perception in typically developing children aged 6–12 with a range of social communication skills, as measured via parent report using the *Social Responsiveness Scale—Second Edition* (SRS-2 [35]), which provides a metric of autism-like traits. Participants were presented with two auditory tokens of /ba/ and attenuated /*a*/ in two conditions. In the audiovisual (AV) condition, a full speaking face was present, producing the articulatory motion of /ba/. In the pixelated (PX) condition, the same face was present; however, the articulatory features of the face (the mouth and jaw) were pixelated. The stimuli were presented within an 80/20 auditory oddball paradigm where */a/* served as the deviant (infrequent) stimuli within both face contexts. Children were required to press a button for the deviant /*a*/ response. The results revealed that both the N100 and P300 responses were modulated by both speech and face conditions. Both the N100 and the P300 effects (deviant waveform–standard waveform) were reduced by the presence of a speaking face, suggesting that neural markers of speech changed with visible articulatory information. Additionally, higher autistic traits, as measured via parent report using the SRS-2, were associated with a reduced P300 effect, regardless of face condition. The authors conclude that reduced sensitivity to subtle changes in phonological information present in human speech may be associated with autism characteristics, such that speech perception deficits may be associated with dimensional approaches of the BAP. 

The current study extends previous work by providing two different listening contexts to examine audiovisual speech perception. Here, we investigate how the neural signature of audiovisual speech perception is modulated within passive (simply listening) and active (differentiating speech stimuli) listening contexts when visual information from the speaking face is present (AV condition) and absent or pixelated (PX condition). First, we hypothesize that an N100 component will be present within both the passive and active experiments and for both AV and PX face conditions, as it is an obligatory auditory response. However, based on the results of Irwin et al. [11] and previous research on AV speech in adults [25,26,27], we expected that in the AV condition, where the full face is present and articulators are visible, there would be an attenuation of the N100 response. In terms of the P300 response, we hypothesize that the passive and active listening contexts will differentially modulate the P300, such that it will be present in the active experiment only (which requires participants to press a button for the deviant response). Additionally, we expect that within the active AV condition, where phonemic restoration is expected to take place, greater neural recruitment (greater P300 amplitude) will be present, given that participants will require greater attention to detect the “deviant” /*a*/ stimulus within the presence of a speaking face, compared to the PX condition where the /*a*/ stimulus may be easier to discriminate. Finally, because previous research from our team has revealed differences in audiovisual speech perception in individuals with ASD and the BAP, we further explore how autism traits are associated with neural markers of AV speech processing, but here for the first time within the adult population. Thus, in addition to comparing neural markers of AV speech perception within AV and PX conditions across passive and active contexts, we will determine the relationship between autism traits measured using the SRS-2 with the N100 and P300 ERP components in this adult sample.

## 2. Materials and Methods

### 2.1. Participants

Forty-one monolingual English adults (27 females, 14 males, mean age = 25.58, SD = 7.94) were recruited for this study. Of the participants, 24 reported their ethnicity as being white, 3 were Asian, 2 were mixed race, 1 was Hispanic, and 11 did not indicate their race/ethnicity. Inclusionary criteria for participation included no known neurological impairment (e.g., no history of epilepsy, recurrent concussions, or neurodegenerative disease). Pure tone audiometry was used to ensure normal hearing. Pure tone air conduction thresholds were screened in both the right and left ears through headphones from 500–4K Hz at 25 dB using a portable Grason-Stadler GSI 18 screening audiometer. Vision acuity screening was also conducted using a multi-letter Snellen Eye Chart. Participants stood 20 feet from the chart which was posted on a wall and were required to read line 8 with both eyes open (aided with glasses, if needed), which is the equivalent of 20/40 vision. 

To further characterize the sample, non-verbal cognitive testing was administered using the *Weschler Abbreviated Scale of Intelligence—Second Edition* (WASI-2) [36]. Testing was conducted within the same session or within a week of the experimental session. To obtain a perceptual reasoning composite, the subtests of block design and matrix reasoning were administered. Most of the participants fell within the average range. Three participants received below-average scores (M = 101.71, SD = 14.88, range = 71–140). 

To collect information on social traits associated with autism, participants completed the *Social Responsiveness Scale—Second Edition* (SRS-2) [35]. The SRS-2 is a self-report form that contains 65 questions (the “Adult Self Report” form was administered) that are graded on a Likert scale. The SRS-2 specifically measures autistic traits in alignment with the Diagnostic and Statistical Manual of Mental Disorders, Fourth Edition [37] and can be used for differential diagnosis. Several subscales are provided within the SRS-2; however, for the purpose of this study, the total score was used as an omnibus measure of autistic traits. The SRS-2 yields a T-score with a mean of 50 and standard deviation of 10. Higher scores on the SRS-2 indicate greater autistic traits. See Table 1 for assessment scores. In addition to these 41 participants, another 7 participants were recruited but ultimately had their data excluded from analyses. The reasons for these exclusions were related to ERP data processing and are detailed below.

### 2.2. Procedures

Participants completed 1–2 sessions lasting approximately 1.5–3 h each, including experimental procedures (eye-tracking and EEG) and behavioral assessments. Eye-tracking data were collected simultaneously with EEG recordings for the purpose of examining other research questions and therefore will not be discussed within the current study. The experiment was created using Experiment Builder software (SR Research Experiment Builder 1.10.165). Recordings took place in a windowless room. The first experiment was passive (required no button press; participants simply viewed the video and listened to the stimuli), and the second experiment was active (participants were instructed to press a button for standard and deviant responses). 

Both passive and active experiments included audiovisual (AV) and pixelated (PX) conditions. In the AV condition, the speaker’s mouth was visible, while in the PX condition, the speaker’s mouth and jaw were obscured by a moving set of pixels, retaining only the outline of a face yet preserving motion. Each condition (AV and PX) contained 200 trials (140 standard and 60 deviant) lasting 2000 ms each. The interstimulus interval (ISI) between trials was 200 ms. Each condition was 9 min and the total experiment time was of 36 min (4 total blocks per condition × 9 min). An auditory oddball paradigm was used to present the stimuli within a 70/30 design; /ba/ was the standard stimulus, occurring 70% of the time and /*a*/ served as the deviant stimulus, 30% of the time. This ratio was present for both conditions and both experiments. The audio was adjusted to a comfortable listening level, which was approximately 65 dB. 

Participants always completed the passive experiment first, followed by the active experiment. For both experiments, the AV condition was first (then the PX condition) to ensure that the phonemic restoration effect was tested without exposure to the contrast of the /ba/ and /*a*/ auditory tokens. Participants were instructed to look at the screen as looks off-screen would pause the experiment, and drift checks were performed at the beginning of each block. During the passive experiment, participants were asked to sit quietly and watch the speaker on the screen. During the active experiment, participants were told to press a button on a game-controller to indicate if they heard a standard or deviant stimulus(separate buttons were used to indicate standard and deviate responses). At the start of the active experiment, the deviant and standard sounds (/*a*/ and /ba/, respectively) were played. This happened before each condition (AV, PX) of the active experiment to remind participants of the deviant stimulus. The buttons were not marked to avoid the potential for participants to look away from the monitor. Participants were required to press a button for both the standard and deviant trials to eliminate a motor confound which may have been present if participants pressed only for the deviant trials. Five practice trials were included at the beginning of each condition and experiment.

### 2.3. ERP Procedures 

A 128-sponge Ag/AgCl electrode high-density sensor array cap (Magstim/EGI, Inc. Whitland, UK) was used to collect electrophysiological data. The cap was soaked for 10 min in a salt-based (KCl) solution to improve conductance. The cap was fitted on the adult’s head using standard procedures outlined by Magstim/EGI [38]. EEG data were recorded using Netstation v. 5.4 software (Magstim/EGI Inc.) The amplifier used was an EGI Net Amps 400 high impedance amplifier. The sample rate was 500 Hz. Impedances were measured before and after each experiment and remained under 40 kΩ.

### 2.4. EEG Stimuli 

Here, we leverage a similar phonemic restoration paradigm used in Irwin et al. [11], yet provide an important addition to the experimental design. Our team has shown substantial differences between children with ASD and neurotypical controls in passive contexts when viewing the speaking face [9] but less marked group differences within an active context where children are asked to press a button when they hear a deviant or less frequent sound, in this case /*a*/ [39]. These findings indicate that task demands may differentially influence audiovisual speech perception. Thus, we use the phonemic restoration paradigm, but include both “passive” (the participant simply looks at the speaking face and listens to the stimuli while the EEG is recorded) and “active” (in which participants are told to press a button when they hear the deviant sound, /*a*/) experiments for each participant. The passive and active experiments mirror different listening contexts that speakers may encounter in their environment. That is, we create two situations in which participants are simply listening to ambient speech (passive) versus a situation in which the environmental demands require the discrimination of speech stimuli (active). The active experiment simulates a context where a listener may be trying to comprehend another speaker’s message.

**Figure 1:**
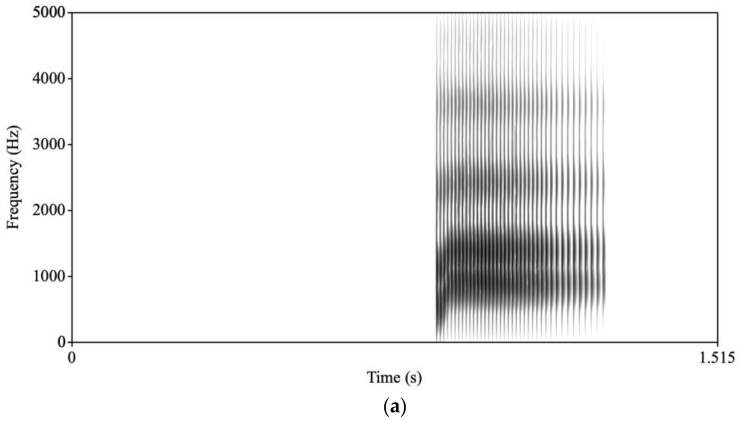

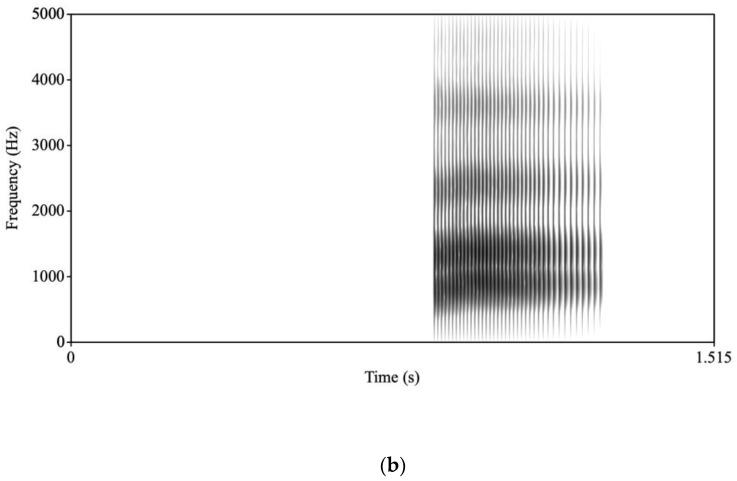
Spectrograms of (**a**) /ba/ and (**b**) /*a*/.

The stimuli used in this study are identical to those in Baron et al. [40]. We included the same speech stimuli in both the passive (participants simply watched the speaking face) and active (participants were asked to press a button for the deviant /*a*/) experiments and these are described in detail below. An adult male speaker was video recorded producing the syllable /ba/ in a sound-attenuated room using a digital video camera approximately 3 feet from the speaker. High-quality audio was captured separately using Praat [41] and a MacBook Pro with a Sennheiser microphone that was placed centrally in front of the speaker approximately 2 feet away below the video camera. /ba/ was repeated for 25 tokens and then a single token was used as the basis for all stimuli. Acoustic parameters were extracted including pitch and amplitude contour, formant trajectories, and voicing for this token. The token had rising formant transitions for F1, F2, and, to a lesser extent, F3, which is characteristic of /ba/. To create the /ba/ stimulus, a new token of /ba/ was synthesized based on these values. To create the /*a*/ stimulus, the synthesis parameters were changed so that onset values were changed for F1 and F2 to reduce the extent of the transitions and lengthen the transition durations for F1, F2, and F3. A new stimulus was then synthesized. For the /ba/, the transitions were 34 ms long and F1 rose from 500 Hz to 850 Hz; F2 rose from 1150 Hz to 1350 Hz; and F3 rose from 2300 Hz to 2400 Hz. The /*a*/ transitions were 70 ms long and F1 rose from 750 Hz to 850 Hz; F2 rose from 1300 Hz to 1350 Hz; and F3 rose from 2300 Hz to 2400 Hz (see Figure 1 for spectrograms of /ba/ and /*a*/). 

**Figure 2:**
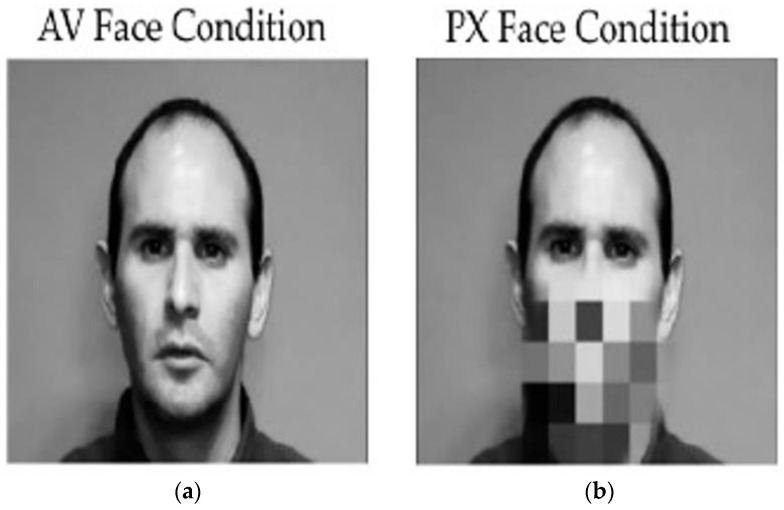
Example of audiovisual (AV) face condition (**a**) where articulators are present and visible and pixelated (PX) face condition (**b**) where pixels cover the articulators. Written informed consent was obtained for the publication of the identifiable image.

The audio stimuli were recorded separately from the video. The /ba/ and /*a*/ synthesized auditory tokens were then oriented to a single frame for accuracy onto the video of the speaker producing /ba/, with the acoustic onsets synchronized with the visible articulation or a video of the face (AV condition). For the PX condition, the video of the mouth was reduced to 36 48 × 48 pixel solid blocks. The mouth region itself was contained within 9 of these blocks (3 × 3) (see Figure 2). All stimulus videos are publicly accessible at https://osf.io/ehvg8/ (accessed on 26 May 2023).

### 2.5. ERP Data Processing

EEG data were analyzed using EEGLAB 2021.0 [42] and ERPLAB 9.0 [43] toolboxes. Preprocessing routines were similar to those used by Irwin et al. (in press) [39] and were applied separately to data from each experiment (active and passive). First, data were sampled at (or, for a small number of participants, downsampled to) 500 Hz. Notch filters were applied at a frequency of 120 Hz (order = 180) to eliminate line noise, and after which the PREP toolbox (v0.55.4) [44] was used to remove additional noise at all line frequencies in the data (60, 120, 180, 240 Hz). The clean_rawdata toolbox (v2.6) was used to identify bad EEG channels via multiple methods that account for channel noise and covariation between channels. This pipeline was used to identify bad electrodes present throughout data collection and these electrodes were replaced using spherical spline interpolation [45]. To automatically identify blinks and lateral eye movements, independent components analysis and the ICLabel algorithm [46] were used to identify components that represented ocular movements in the EEG data with at least 80% probability. These components were removed prior to subsequent analysis. Additional ocular independent components were identified manually for a small number of participants. Data were then subjected to a band-pass filter from 0.3–30 Hz (Butterworth filter, 12 db./oct roll-off) and re-referenced to the average reference (the vertex reference, Cz, was used during recording). A total of 900 ms epochs were created which included a 100 ms pre-stimulus baseline and an 800 ms post-stimulus interval. Data were then baseline-corrected using the mean of the pre-stimulus window [47].

For artifact rejection, we measured the horizontal electrooculogram (HEOG) as the difference between channels 125 and 128. We measured the vertical electrooculogram (VEOG) according to four pairwise differences between channels 126 and 8, 126 and 14, 127 and 21, and 127 and 25. Epochs containing a lateral eye movement were rejected if HEOG exceeded |μV| ≥ 55 and rejected for containing a blink if VEOG exceeded |μV| ≥ 150, within sliding 80 ms windows. These artifact rejection criteria were applied between −100 and 100 ms. Ocular independent components were then removed, and data were re-referenced to the average reference (the vertex reference, Cz, was used during recording). The criteria were then applied to the entire window. (As the visual stimulus during speech onset affected the perceived sound, this ensured that trials were always rejected if participants made eye movements during the critical window.) Finally, for all participants, each EEG channel was marked as bad—and interpolated from nearby channels—in every epoch in which it varied by more than 200 μV (max.–min.). Channels that were marked as bad in at least 33% of epochs were marked as bad (and interpolated) in all epochs (for 96% of participant/experiment conditions, no channels were interpolated in this way at this stage). Epochs that contained at least 20 bad channels were discarded from analysis. Non-rejected trials were averaged within each condition for subsequent analysis.

For statistical analyses, all preserved deviants were used, and only the standard that occurred before the deviant was used. This was the case to keep the trial counts consistent for both the standards and deviants. Therefore, 60 possible trials for every combination of experiment (active and passive), face context condition (AV and PX), and speech sound (standard and deviant) were included in the analyses. All participants had at least 29 (72.5%) usable trials in each condition (the threshold for inclusion was 20 or 50%), and had on average 55 usable trials in each condition (53–58 usable trials across different conditions).

As noted above, 7 additional participants were excluded from analyses for various reasons: A recording issue that resulted in impedance measurements being collected during 50% of trials in one experiment (*n* = 1), too few usable trials in one or more conditions (*n* = 2), or too many channels interpolated (>20 or >15.5%) in one or more experiments (*n* = 2). In addition, two participants who otherwise met the inclusion criteria were excluded due to having highly irregular/extreme global field power (GFP) measurements in one or more conditions, which suggested that the processing pipeline was ill-suited to their data.

### 2.6. Analysis 

**Figure 3:**
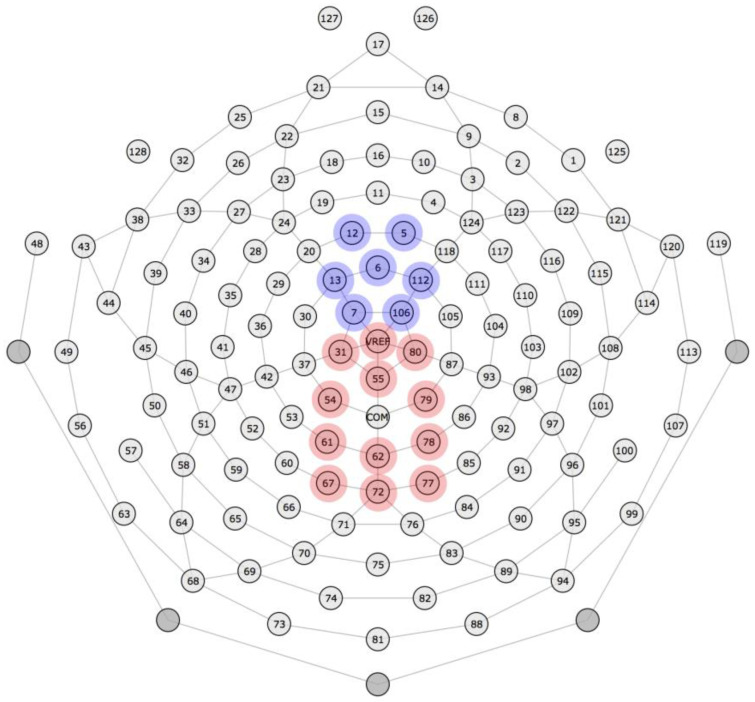
Electrode montage. Electrodes contributing to the N100 component cluster are highlighted with blue circles. Electrodes contributing to the P300 component cluster are highlighted with red circles.

Grand mean waveforms were visually inspected to determine where the N100 and P300 components were observed. Both components had typical spatiotemporal distributions (see Figure 3 for the electrode montage): The N100 was identified in a cluster of 7 electrodes in the frontocentral region from approximately 50 to 150 ms, with maximal component amplitude from approximately 75 to 125 ms. The P300 was identified in a cluster of 12 electrodes in the medial–parietal region from approximately 250 to 700 ms, with maximal component amplitude from approximately 350 to 550 ms. Accordingly, amplitudes on each trial were averaged across these electrodes and maximal time windows (N100: 7 electrodes, 75–125 ms; P300: 12 electrodes, 350–550 ms), yielding one N100 amplitude and one P300 amplitude for each trial.

*R* software (v. 4.2.2; R core team, 2022. [48] was used for analyses. Linear mixed-effects models (the *lme4* package, v. 1.1-31) [49] using single-trial amplitudes were conducted. For each component, data from all conditions were analyzed using a single model with three factors: context (two levels: passive = −0.5, active = +0.5), condition (two levels: pixelated (PX) = −0.5, audiovisual (AV) = +0.5), and stimulus (two levels: standard /ba/ = −0.5, deviant /*a*/ = +0.5). The models included these factors, as well as all interactions between them, as fixed effects, as well as the maximal random effects structure supported by the data. To identify the maximal random effects structure, we followed a three-step procedure (for these models and all other supplementary models). The bobyqa optimizer was used to fit a model with a maximal random effects structure: random intercepts for participants, all within-factor random slopes and their interactions, and correlations between random slopes. If no convergence was present within a model, we removed correlations between random slopes. If the resulting model still did not converge, we identified random slopes accounting for less than 1% of the variance of their associated random factors, and then removed all such slopes simultaneously [50], which always resulted in convergence. The Satterthwaite method in the lmerTest package (v. 3.1-3) [51] was used to estimate degrees of freedom. Follow-up contrasts were applied to the fitted model for significant interactions using the emmeans package (v. 1.8.4-1) [52]. To check whether our results were dependent on the analytic approach we employed, we conducted two additional sets of analyses. The first used the same approach as the primary models, but included a (within-condition) trial number and its interactions as additional factors (both for fixed and random effects). The second approach employed repeated-measures ANOVAs instead of mixed-effects models: P300 amplitudes were averaged for each participant across all trials in the same condition, and after which participant means were analyzed using a three-way ANOVA with context, condition, and stimulus as factors. For both components, both approaches yielded identical patterns of statistical significance to the primary approach, and so we do not discuss them further.

For the active experiment, response accuracy for /ba/ or /*a*/, as measured via accuracy of button press, was similarly analyzed using a generalized mixed-effects model with a logit-link function and binomial distribution, with accuracy as the dependent variable (1 = correct, 0 = incorrect). The model included fixed effects of condition, or mouth visibility (two levels: pixelated (PX) = −0.5, audiovisual (AV) = +0.5) and stimulus (two levels: standard /ba/ = −0.3, deviant /*a*/ = +0.7); all interactions between these fixed effects; and a maximal random effects structure (the model converged with random correlations and all random effects were included). All participants in the EEG analyses were included in the accuracy analyses except for one participant whose data contained no button presses due to an equipment malfunction; a small number of other trials with missing responses (0.6%) were also excluded.

## 3. Results

### 3.1. ERP Analyses

**Figure 4:**
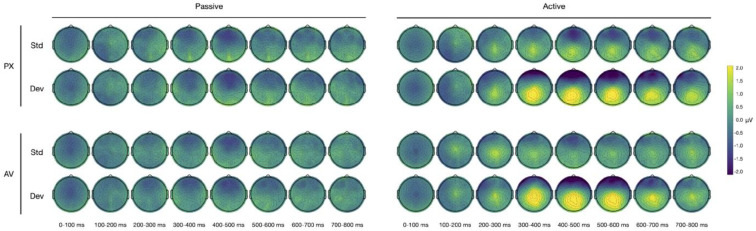
ERP scalp maps for all conditions.

The scalp maps for all of the conditions are shown in Figure 4.

### 3.2. N100

**Figure 5:**
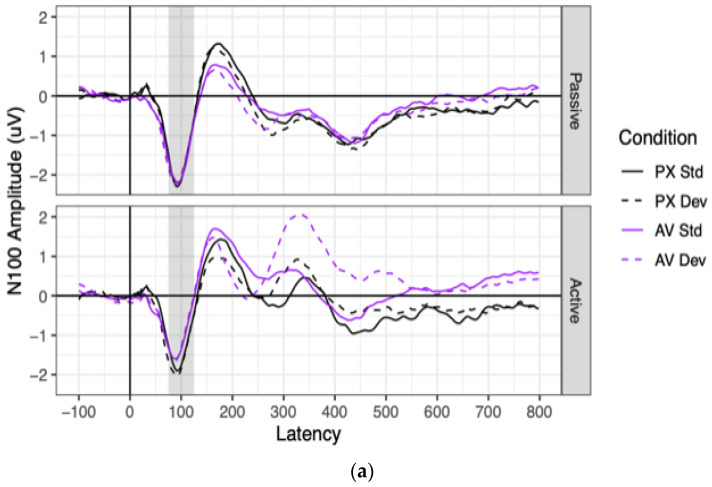

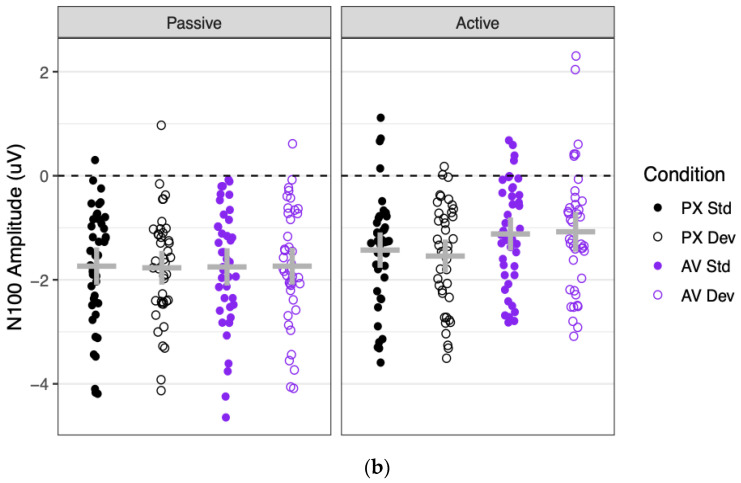
N100 ERPs in the frontocentral electrode cluster of (**a**) grand mean waveforms and (**b**) averaged amplitudes. (**a**) Grand mean waveforms for all conditions in the frontocentral electrode cluster. The time window over which the N100 component was averaged (75–125 ms) is highlighted in gray. (**b**) N100 amplitudes for all participants and conditions, averaged over the frontocentral electrode cluster and the highlighted time window. Each point represents one participant’s mean condition. Gray horizontal lines represent sample means; gray vertical lines represent 95% confidence intervals.

Grand mean waveforms and component amplitudes are shown for the N100 in Figure 5. Effect sizes and significance tests for all predictors are shown for the N100 in Table 2.

Significant N100 ERPs were observed in every combination of context, condition, and stimulus (all means < −1.08, all *ts* < −6.33, all *p* < 0.001). N100 amplitudes were significantly more negative in the passive context (mean = −1.75 μV) than in the active context (mean = −1.29 μV), as indicated by a main effect of context. N100 amplitudes were also marginally more negative to PX stimuli (mean = −1.62 μV) than to AV stimuli (mean = 1.42 μV), as indicated by a marginally significant effect of condition. This marginal effect of condition was driven entirely by the active context, as shown by a significant two-way interaction between context and condition. Contrasts showed that in the passive context, N100 amplitudes did not differ between the PX condition (mean = −1.75 μV) and the AV condition (mean = −1.75 μV; difference: *B* = 0.01 μV, SE = 0.13 μV, *t*(79) = 0.08, *p* = 0.940). In the active context, however, N100 amplitudes were more negative to PX stimuli (mean = 1.48 μV) than to AV stimuli (mean = −1.10 μV; difference: *B* = 0.38 μV, SE = 0.13 μV, *t*(77) = 2.91, *p* = 0.005). All effects involving stimulus (the main effect and all interactions) were non-significant.

### 3.3. P300 

**Figure 6:**
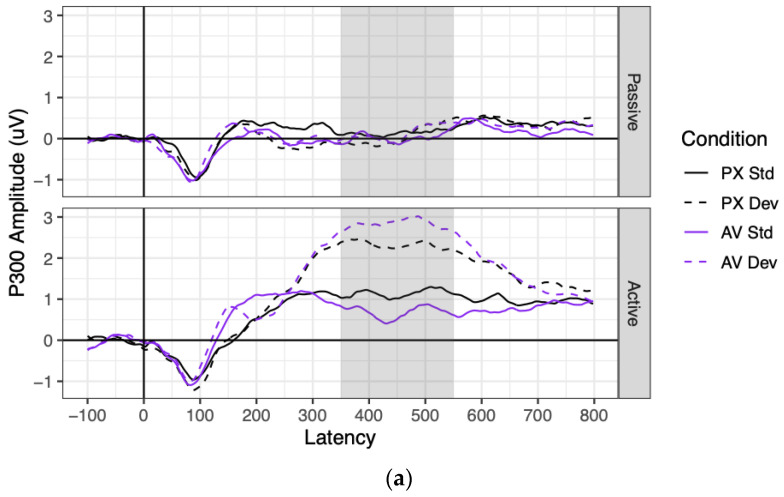

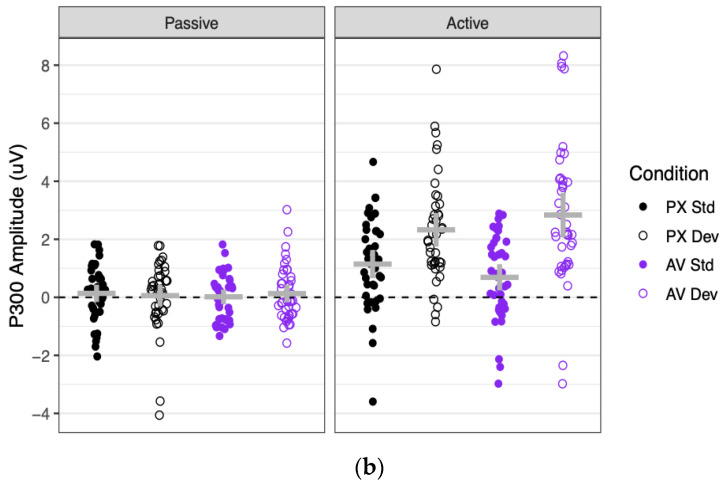
P300 ERPs in the medioparietal electrode cluster of (**a**) grand mean waveforms and (**b**) averaged amplitudes. (**a**) Grand mean waveforms for all conditions in the medioparietal electrode cluster. The time window over which the P300 component was averaged (350–550 ms) is highlighted in gray. (**b**) P300 amplitudes for all participants and conditions, averaged over the medioparietal electrode cluster and the highlighted time window. Each point represents one participant’s mean condition. Gray horizontal lines represent sample means; gray vertical lines represent 95% confidence intervals.

Grand mean waveforms and component amplitudes are shown for the P300 in Figure 6. Effect sizes and significance tests for all predictors are shown for the P300 in Table 3.

P300 amplitudes were significantly more positive in the active context (where they significantly differed from 0; mean = 1.75 μV, SE = 0.18 μV, *t*(74) = 9.96, *p* < 0.001) than in the passive context (where they did not significantly differ from 0; mean = 0.09 μV, SE = 0.18 μV, *t*(75) = 0.51, *p* = 0.612), as indicated by a main effect of context. P300 amplitudes were also significantly more positive to deviant stimuli (1.34 μV) than to standard stimuli (0.50 μV), as indicated by a main effect of stimulus. These factors significantly interacted: P300 amplitudes were significantly more positive to deviant than standard stimuli in the active context (*B* = 1.67 μV, SE = 0.16 μV, *t*(76) = 10.13, *p* < 0.001), but not in the passive context (*B* = 0.03 μV, *SE* = 0.17 μV, *t*(80) = 0.16, *p* = 0.875).

As for the effects of condition, the PX and AV conditions did not significantly differ in P300 amplitude, nor did the effect of condition vary by context. However, the effect of stimulus (deviant–standard) was twice as large in the AV condition (*B* = 1.14 μV) as in the PX condition (*B* = 0.55 μV), as represented by a significant interaction between condition and stimulus. This interaction was driven entirely by the active context, as shown by a marginally significant three-way interaction between context, stimulus, and condition. Contrasts showed that the effect of stimulus (deviant–standard) was non-significant in the passive context for both the PX and AV conditions (both |*B*| < 0.11 μV, both *p* > 0.61), which did not significantly differ from each other (*B* = 0.08 μV, SE = 0.14 μV, *t*(108) = 0.58, *p* = 0.562). In contrast, the effect of stimulus was significant in the active context both for the PX condition (*B* = 1.16 μV, SE = 0.22 μV, *t*(163) = 5.39, *p* < 0.001) and the AV condition (*B* = 2.17 μV, SE = 0.21 μV, *t*(159) = 10.12, *p* < 0.001), and it was almost twice as large in the AV condition as the PX condition (*B* = 0.50 μV, SE = 0.14 μV, *t*(102) = 3.63, *p* < 0.001).

### 3.4. Relationships with SRS-2 Scores

To determine whether the SRS-2 scores (as independent variables) were related to P300 amplitudes (as the dependent variable), we conducted an additional analysis that included the SRS-2 total score and its interactions with other factors, as fixed effects. For this analysis, we discarded the data from two participants: one participant who did not complete the SRS-2, and one participant whose SRS-2 score was more than three standard deviations higher than the mean (75, where all other participants had scores between 38 and 61).

### 3.5. N100

Relationships between SRS-2 scores and N100 amplitudes in each condition are shown in Supplementary Figure S1. SRS-2 scores were not associated with N100 amplitudes: all effects involving SRS-2 scores were not significant (all *p* > 0.20).

### 3.6. P300

**Figure 7:**
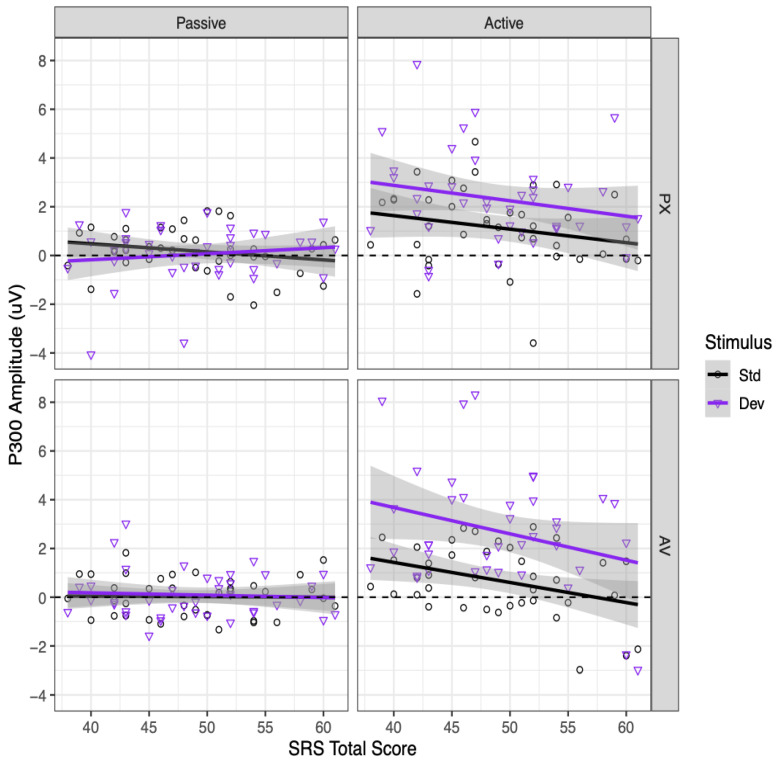
Associations between P300 amplitudes and SRS-2 scores. Each point represents one participant’s average component amplitude in one condition. Error ribbon = 95% CI. Correlations shown for visualization purposes only; significance testing was performed using mixed-effects models.

Relationships between SRS-2 scores and P300 amplitudes in each condition are shown in Figure 7. Participants with higher SRS-2 scores had marginally less positive P300 amplitudes overall, as indicated by a marginally significant main effect of SRS-2 score, *B* = −0.041, SE = 0.023, *t*(37) = −1.79, *p* = 0.082. This effect was qualified by a significant interaction between SRS-2 score and context, *B* = −0.072, SE = 0.034, *t*(37) = −2.10, *p* = 0.042. Higher SRS-2 scores were significantly associated with less positive P300 amplitudes in the active context, *B* = −0.077, SE = 0.029, *t*(68) = −2.69, *p* = 0.009, but not in the passive context, *B* = −0.005, SE = 0.029, *t*(68) = −0.17, *p* = 0.865. Further exploring the relationship between SRS-2 scores and P300 amplitudes in the active context, this relationship was significant for the active AV condition, *B* = −0.094, *SE* = 0.033, *t*(111) = −2.85, *p* = 0.005, and marginally significant for the active PX condition, *B* = −0.059, SE = 0.033, *t*(111) = −1.79, *p* = 0.076. No other effects involving the SRS-2 total score were significant (all *p* > 0.15), including all interactions with stimulus.

### 3.7. Response Accuracy 

Additionally, button press data for the active experiment were collected and analyzed, which revealed accuracy for discrimination of the deviant response in the active context for AV and PX conditions. Effect sizes and significance tests for all predictors are shown in Table 4; F1 means are provided below for interpretability.

Button press accuracy was close to ceiling for the PX condition, as participants correctly identified 96.7% of the /ba/ tokens and 97.4% of the /*a*/ tokens. However, in the active AV condition, where phonemic restoration was expected to take place, participants correctly identified 99.5% of the /ba/ tokens but only 67.6% of the /*a*/ tokens. This was supported by a significant interaction between condition and stimulus. Contrasts showed that accuracy was lower to /*a*/ stimuli in the active AV condition than to stimuli in any other active condition (all *B* < −3.97, all *z* < −6.86, all Tukey-corrected *p* < 0.001), and also that accuracy was *higher* for /ba/ stimuli in the active AV condition than in the active PX condition (*B* = 1.51, SE = 0.50, *z* = 3.02, Tukey-corrected *p* = 0.013). This suggests a visual influence on perception in the AV condition relative to the PX condition, with the visual /ba/ leading to greater auditory /ba/ perception for all stimuli, including /*a*/ stimuli, which were perceived as /ba/ due to phonemic restoration. For a more detailed analysis of the button press data, please see Baron et al. [40]. The participant sample in Baron et al. (2023), which excluded participants and/or trials based on eye-tracking measures rather than ERP measures, differed slightly from the current sample (90% overlap between samples). As a result, the summary statistics reported there differ slightly from those reported here

## 4. Discussion

We investigated the neural mechanisms of audiovisual speech perception in adults using a phonemic restoration paradigm within two experimental contexts (passive and active) where the articulators were visible (AV condition) and absent (PX condition). We expected phonemic restoration to take place in the AV condition where the visible articulation of phonemic stimuli by the mouth “influences” the missing initial consonant for the deviant stimuli (/*a*/), allowing the participants to perceive /ba/. The passive experiment (participant simply viewed a speaking face) and active experiment (participant actively pressed a button to identify stimuli) created a model of naturalistic listening situations where listeners were tasked with discriminating changes in speech. We hypothesized that both experimental context and face condition would differentially modulate the neural mechanisms associated with AV speech processing (N1 and P300 components). Specifically, the presence of a speaking face would attenuate the N100 component regardless of the experimental context (passive or active), and the P300 response would be present within the active experiment only for both face conditions (AV and PX), with greater amplitude for the AV condition. Furthermore, we explored the relationship between the neural indices of audiovisual speech perception and social communication behaviors measured by the SRS-2 to determine the extent to which autistic traits were associated with the neural processes related to AV speech perception in the BAP. 

In terms of the electrophysiological data, we observed an early N100 component in all of the experimental contexts (passive/active) and conditions (AV/PX). The N100 was modulated by experimental context, where the N100 was significantly more negative in the passive context than in the active context; however, this effect was largely driven by the condition. The N100 was significantly more negative in the *active* experiment for the PX condition compared to the AV condition. Put differently, the N100 in the active AV condition was attenuated compared to the active PX condition, but differences between face contexts were not present within the passive experiment. This finding is consistent with several studies, demonstrating that the visual presence of the articulators attenuates the N100 response in adults [25,26,27], but also extends previous findings by demonstrating that the N100 response may be attenuated in the presence of visible articulation while modulated by factors such as attention to task. This aligns with the audiovisual speech perception literature that has demonstrated changes in electrophysiological markers of speech perception due to different listening conditions or attentional load [53,54]. 

Consistent with our hypothesis, the P300 response was present within the active experiment, for both the AV and PX conditions, but not in the passive experiment. Within the active experiment, the P300 effect (deviant–standard) was significantly greater in the AV condition compared to the PX condition, as indicated by a marginal interaction between context, condition, and stimulus. This may be an indication of experimental task complexity, as the influence of the visual speaking face within the AV condition required greater attention from the participants to identify the deviant /*a*/ stimuli. This is consistent with the literature, suggesting that the P300 amplitude is influenced by the task complexity, where greater amplitude for the deviant stimuli may reflect increased processing demands [55]. The behavioral results of the button press accuracy further corroborated the electrophysiological findings. Within the active experiment, participants demonstrated higher accuracy in identifying the deviant /*a*/ stimuli in the PX condition, where participants were not influenced by the visual features of the articulators (lips, tongue, jaw). Within the AV condition, participants were only ~64% accurate at correctly identifying the /*a*/ deviant, suggesting a visual phonemic restoration effect for the /*a*/ stimuli within the active AV condition.

Importantly, our analyses also revealed a negative correlation between the P300 responses in the active experiment and the total score on the SRS-2 [35]; hence, lower amplitudes for the P300 component in the active experiment were associated with higher autistic traits. This relationship was statistically significant only for the active AV condition, although it was not significantly stronger than in the active PX condition. There was no association between the SRS-2 scores and the N100 ERP component. To the authors’ knowledge, this is the first study to associate neural indices of audiovisual speech perception with autistic traits within the BAP in adults. These results are also consistent with our prior research on audiovisual speech perception within the BAP in children. Irwin et al. [11] demonstrated that higher autistic traits as measured using the SRS-2 were correlated with a reduced P300 effect within both the AV and PX face conditions using a similar phonemic restoration paradigm. Also, there was no association between the N100 and SRS-2 scores. This work aligns with previous studies that suggest that children with ASD may have difficulties in processing subtle changes in speech stimuli [56]. Given that the association between the P300 and the SRS-2 scores is present in both the AV and PX conditions, it is possible that difficulties in speech perception more generally, or attention to subtle changes in phonemic or linguistic stimuli, are associated with the BAP, independent of visual speech. Further work is necessary to determine whether impairments in speech processing and/or audiovisual speech perception are associated with the BAP.

It is also important to note that the P300 ERP response has been associated with attentional processing [29] as well as phonological working memory within auditory tasks [57,58]. There is evidence to suggest that first-degree relatives of autistic children demonstrate differences in phonological working memory compared to healthy controls [59,60,61]. Both studies revealed that parents of children with ASD performed significantly lower on tasks of phonological working memory, specifically non-word repetition, when compared to controls. Furthermore, McFadden et al. [59] and Wilson et al. [61] demonstrated neural differences in the parents of children with ASD during phonological processing tasks, and these differences were consistent with patterns in ASD probands. Considering our current findings, these differences in the neural mechanisms associated with the processing of language, and their cognitive associations, may be distinct neurophysiological biomarkers of ASD. Further work is necessary, specifically in first-degree relatives of children with ASD, to determine the extent to which these neurophysiological biomarkers of language may give rise to research surrounding the heritability of ASD.

This study adds to the audiovisual literature by replicating findings in which the presence of visible articulatory speech influences both behavioral speech perception and the neural mechanisms which underlie audiovisual speech processing in neurotypical adults [62,63,64]. It is also an important step in establishing a relationship between the neural mechanisms associated with audiovisual speech perception and social pragmatic functioning within the BAP in an adult sample. Importantly, our conclusions should not be extended beyond the populations from which we sampled. There are other factors which may impact audiovisual speech perception, such as age [65,66,67,68,69]; therefore, future research using different adult populations would be an important next step to establish the generalizability of our findings. Also, the stimuli used for this study consisted of syllables. Future studies on audiovisual speech perception within the BAP may consider the use of word-level speech, which may provide another element of naturalistic listening conditions.

## 5. General Conclusions

We investigated the neural indices of audiovisual speech perception using a phonemic restoration paradigm which included passive and active listening contexts to determine its association with autism traits within the BAP. The obligatory N100 ERP component was present within all listening and face contexts; however, the N100 response was significantly attenuated in the active AV condition, where visible articulation was present. The P300 response was present within the active experiment only, for both AV and PX conditions; however, amplitudes were significantly greater in the AV condition where phonemic restoration was expected to take place. Finally, autistic traits measured via self-report using the SRS-2 were negatively correlated with the P300 response, such that higher autistic traits were associated with generally smaller P300 responses within the active context, and especially in the active AV condition. Atypical neural indices of an audiovisual speech perception may be a biomarker of the BAP. Further studies are necessary to determine the extent to which atypical audiovisual speech perception is associated with the BAP. 

## Figures and Tables

**Table 1:** Demographic characteristics.

Assessment	N	Mean (SD)	Range
Age	41	25.58 (7.94)	19–49.67
WASI PR Composite Score	41	101.71 (14.88)	71–140
SRS-2 Total	40	49.53 (7.46)	38–75

Note: WASI PR = Weschler Abbreviated Scale of Intelligence, Second Edition, Perceptual Reasoning Index, SRS-2 = Social Responsiveness Scale—Second Edition, Adult Self Report.

**Table 2:** Results of single-trial analyses of N100 amplitude.

Effect	* B * (μV)	SE (μV)	dfs	*t*	*p*
**Intercept**	**−1.52**	**0.14**	**40**	**−11.05**	**<0.001**
** Context (Passive vs. Active) **	**0.46**	**0.10**	**40**	**4.49**	**<0.001**
* Condition pixelated vs. audiovisual *	*0.20*	*0.10*	*40*	*2.00*	*0.052*
Stimulus (Standard vs. Deviant)	−0.02	0.07	39	−0.35	0.725
Context * Condition	**0.37**	**0.18**	**39**	**2.12**	**0.040**
Context * Stimulus	−0.04	0.11	17,995	−0.31	0.756
Condition * Stimulus	0.11	0.11	17,981	0.95	0.343
Context * Condition * Stimulus	0.09	0.23	17,987	0.42	0.677

Note. For each factor, a positive *B* represents a more positive (less negative) N100 amplitude to the second factor level specified (Active, AV, Deviant). Denominator degrees of freedom (dfs) were estimated via the Satterthwaite method; all effects lacking a corresponding random slope (here, all three interactions involving stimulus) had atypically large dfs, though this did not affect statistical significance relative to other dfs. Effects that were statistically significant (*p* < 0.05) are highlighted in **bold**; effects that were marginally significant (0.05 < *p* < 0.10) are *italicized*. * means “by”.

**Table 3:** Results of single-trial analyses of P300 amplitude.

Effect	* B * (μV)	SE (μV)	dfs	*t*	*p*
** Intercept **	** 0.92 **	** 0.14 **	** 40 **	** 6.56 **	** <0.001 **
** Context (Passive vs. Active) **	** 1.66 **	** 0.21 **	** 40 **	** 7.81 **	** <0.001 **
Condition (PX vs. AV)	0.00	0.16	41	0.01	0.991
** Stimulus (Standard vs. Deviant) **	** 0.85 **	** 0.13 **	** 41 **	** 6.57 **	** <0.001 **
Context * Condition	0.07	0.28	40	0.23	0.817
Context * Stimulus	** 1.64 **	** 0.21 **	** 40 **	** 7.90 **	** <0.001 **
Condition * Stimulus	** 0.58 **	** 0.18 **	** 17,959 **	** 3.32 **	** <0.001 **
Context * Condition * Stimulus	* 0.84 *	* 0.43 *	* 38 *	* 1.94 *	* 0.060 *

Note: For each factor, a positive *B* represents a more positive P300 amplitude to the second factor level specified (Active, AV, Deviant). Denominator degrees of freedom (dfs) were estimated via the Satterthwaite method; all effects lacking a corresponding random slope (here, the interaction of Condition * Stimulus) had atypically large dfs, though this did not affect statistical significance relative to other dfs. Effects that were statistically significant (*p* < 0.05) are highlighted in **bold**; effects that were marginally significant (0.05 < *p* < 0.10) are *italicized*. SE, Standard Error. * means “by”.

**Table 4:** Results of single-trial analyses of button press responses in the active context.

Effect	* B *	SE	*z*	*p*
** Intercept **	** 5.46 **	** 0.33 **	** 16.64 **	** <0.001 **
Condition (PX vs. AV)	−0.12	0.39	−0.31	0.756
** Stimulus (Standard vs. Deviant) **	** −2.77 **	** 0.47 **	** −5.85 **	** <0.001 **
Condition * Stimulus	** −5.43 **	** 0.75 **	** −7.27 **	** <0.001 **

Note: For each factor, a positive *B* represents greater accuracy in the second factor level specified (AV, Deviant). As the binomial model used a logit link function, *B* and SE values are given in log-odds space. Effects that were statistically significant (*p* < 0.05) are highlighted in **bold**.

## Data Availability

The data supporting all statistical analyses in this article are publicly available. These data can be found here: https://osf.io/7xguk/?view_only=58c5dc1ba36d49849fd2da678b258604.

## Supplementary Materials

The following supporting information can be downloaded at https://www.mdpi.com/article/10.3390/brainsci13071011/s1: Figure S1: Non-significant associations between N100 amplitudes and SRS-2 scores. Each point represents one participant’s average component amplitude in one condition. Error ribbon = 95% CI. Correlations shown for visualization purposes only; significance testing was performed using mixed-effects models.
