# Supplementary material for: Event-Related Potentials in Assessing Visual Speech Cues in the Broader Autism Phenotype: Evidence from a Phonemic Restoration Paradigm
Source: Brain Sci. 2023 Jun 30;13(7):1011. doi: 10.3390/brainsci13071011 (PMC10377560; doi:10.3390/brainsci13071011)
Supplement: Supplementary file 1 [file brainsci-13-01011-s001.zip › brainsci-2445907-supplementary-done.pdf]

brain sciences

MDPI

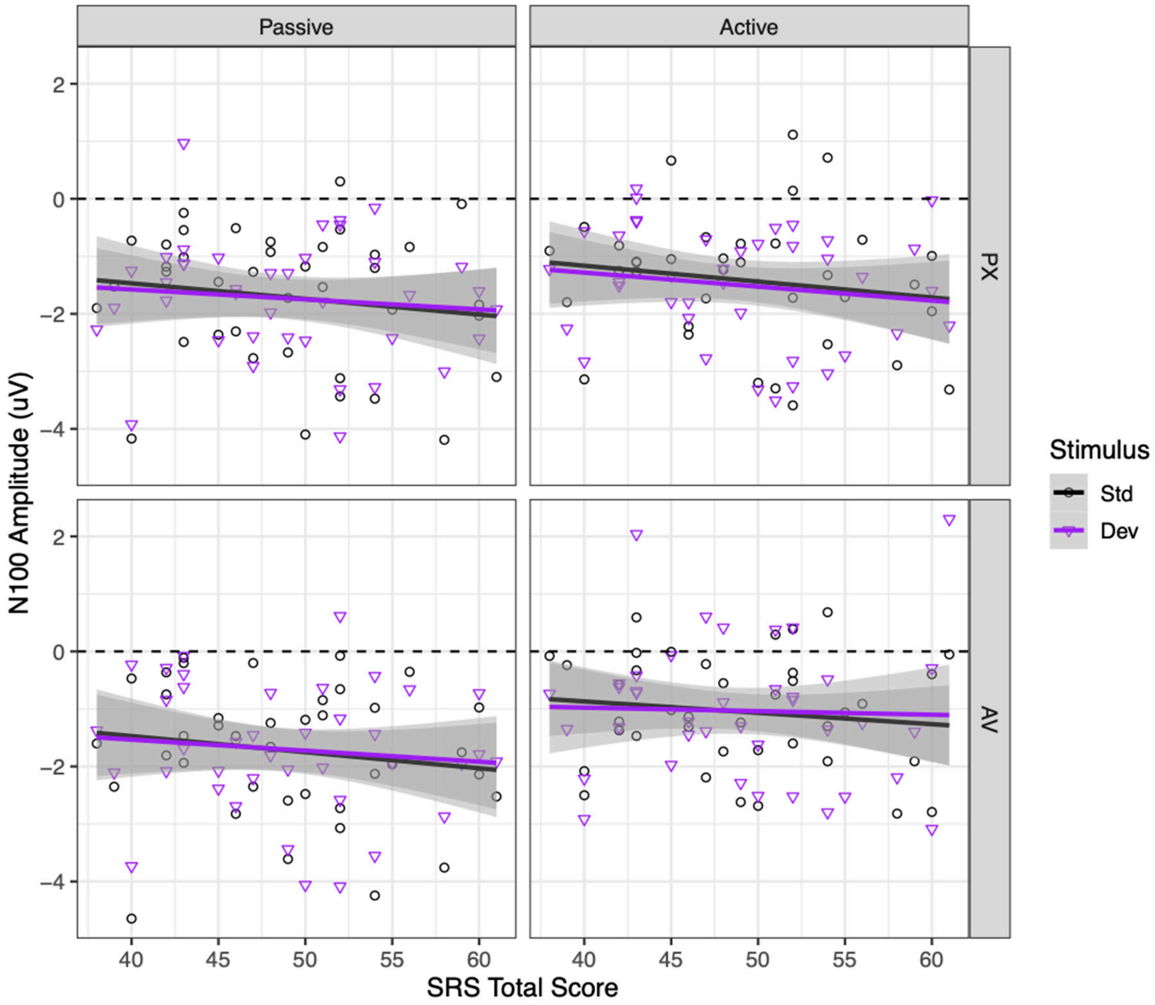

Figure S1. Non-significant associations between N100 amplitudes and SRS-2 scores. Each point represents one participant's average component amplitude in one condition. Error ribbon = 95% CI. Correlations shown for visualization purposes only; significance testing was performed using mixed-effects models.
